# Complete excision of a large pancreatic neuroendocrine neoplasm

**DOI:** 10.11604/pamj.2018.31.240.14942

**Published:** 2018-12-20

**Authors:** Rudolph Darko, Frank Edwin, Jehoram Anim

**Affiliations:** 1 Department of Surgery, School of Medicine & Dentistry, University of Ghana, Accra, Ghana; 2 Department of Surgery, School of Medicine, University of Health & Allied Sciences, Ho, Ghana; 3 Cellular Pathology Laboratory, Forensic Department, Ghana Standards Authority, Accra, Ghana

**Keywords:** Pancreas, neuroendocrine, carcinoma, synaptophysin

## Abstract

Pancreatic neuroendocrine tumours localized to the pancreas and amenable to complete surgical resection are rarely reported. In West Africa, such patients present too late for surgery to be considered. In the reported case, a patient with persistent epigastric pain underwent a computed tomographic examination which led to the discovery of a large (6cm x 5cm) localized tumour in the body and tail of the pancreas. Complete resection of the tumour was performed. Histological examination showed a pancreatic neuroendocrine tumour without capsular invasion. Adjuvant chemotherapy was deemed unnecessary. The patient remains symptom free 2 years after the procedure with no evidence on subsequent imaging of tumour recurrence. Although extremely rare, large pancreatic neuroendocrine tumours may still be amenable to complete excision.

## Introduction

Resectable tumours are rarely encountered in the West African sub-region. Even more uncommon is the neuroendocrine neoplasm of pancreatic origin presenting early enough for surgical exploration. We recently managed a middle-aged woman who presented to a peripheral facility with persistent epigastric pain of recent onset. Her insistence on complete understanding of the source of the pain led to a computed tomographic examination and discovery of a large tumour in the body and tail of the pancreas. She underwent exploratory laparotomy and complete resection of the tumour which proved on histological examination to be a neuroendocrine neoplasm. Her postoperative recovery was uneventful and she remains disease-free 2 years after the procedure.

## Patient and observation

**Figure 1:**
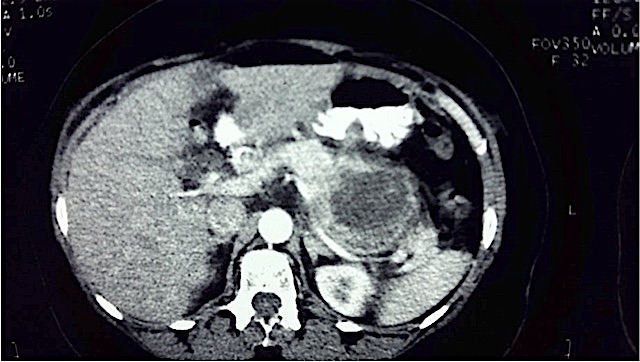
Axial abdominal contrast-enhanced computed tomographic scan showing a large tumour in the region of the body of the pancreas

**Figure 2:**
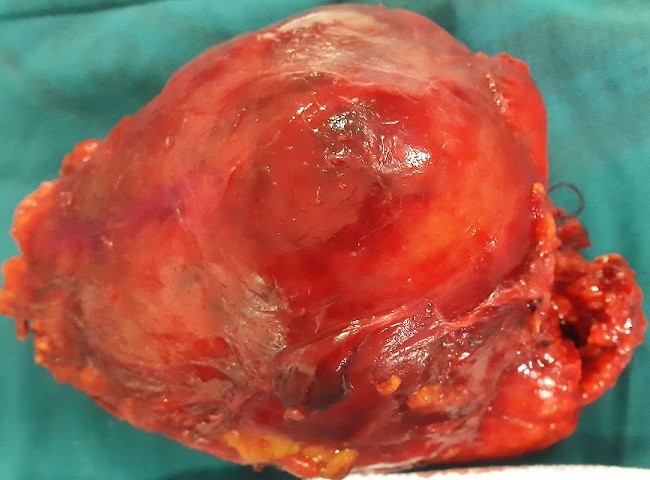
Tumour resected from the body and tail of the pancreas

A 57 year old lady presented to a peripheral hospital with complaints of penetrating epigastric pain of a week’s duration. There was no vomiting or weight loss. Bowel movements were normal. Clinical examination at the time was unrevealing. An abdominal ultrasound examination was reported as normal. The patient’s insistence on full investigation of the persistent epigastric pain led to a computed tomographic examination and discovery of a pancreatic tumour subsequent to which she was referred to our institution. The CT scan (Figure 1) showed a large tumour (6cm in largest diameter) in the body and tail of pancreas with no evidence of metastasis. She was prepared for exploratory laparotomy. The pancreas was explored through the gastrocolic omentum. The body and tail of the pancreas including the tumour were resected (Figure 2). The spleen was also removed. The end of the pancreas was suture ligated. Her post-operative recovery was uneventful.

**Figure 3:**
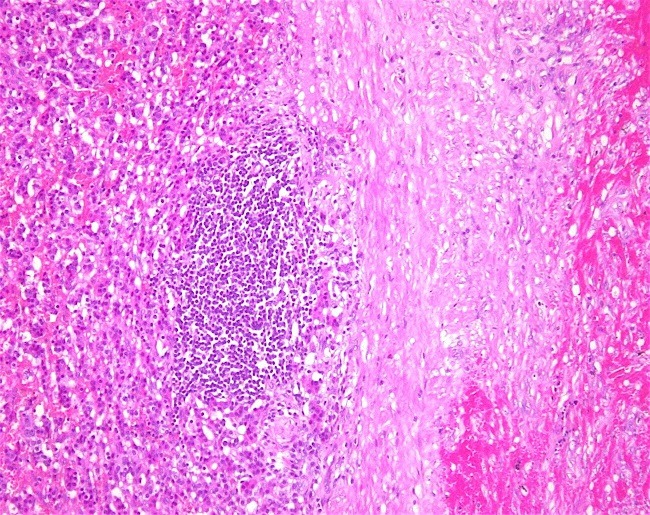
Histological examination of the tumour showed a well circumscribed mass (center)

**Figure 4:**
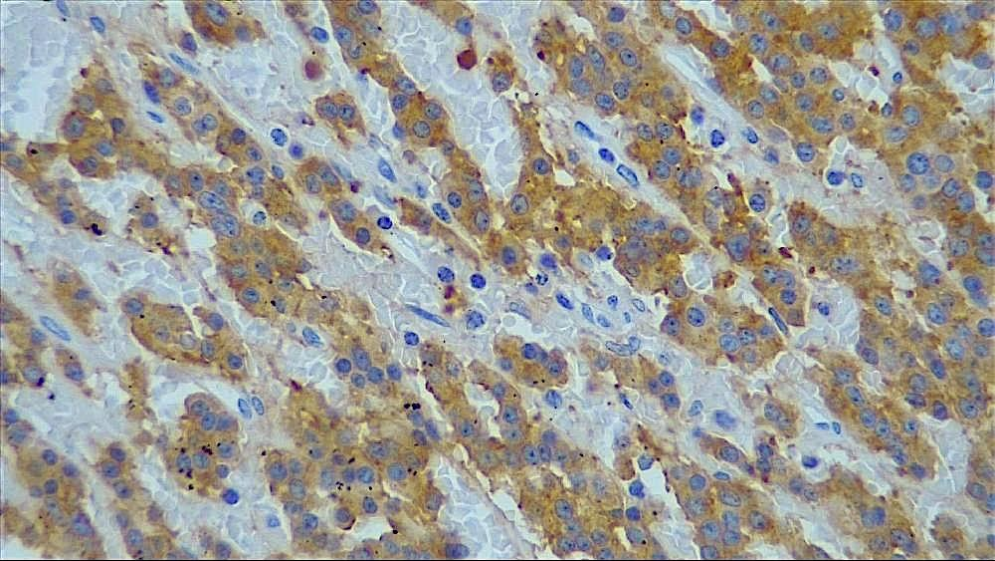
The synaptophysin stain was strongly positive

**Figure 5:**
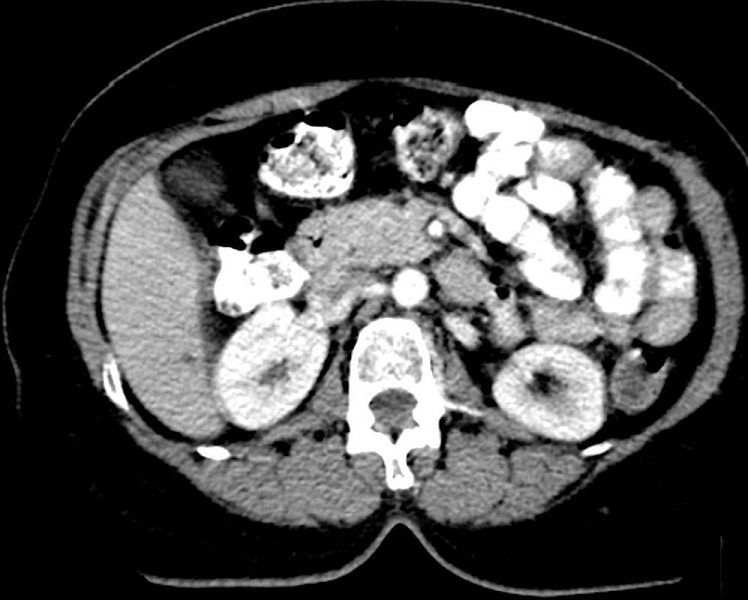
Axial computed tomographic scan performed two years after resection showed no evidence of tumour recurrence

Histological examination of the specimen (Figure 3) showed a well circumscribed ovoid tumour 60mm x 50mm across. Sections revealed islands and trabeculae of regular cells with variable amounts of eosinophilic cytoplasm, mostly regular nuclei with indistinct nucleoli. Areas of necrosis were present with hemorrhage. A fibrous pseudo-capsule was noted around the tumour and the resection of the tumour appeared complete. No capsular of vascular invasion was seen. Immuno-peroxidase staining was positive for chromogranin A. Synaptophysin was strongly positive (Figure 4), CD10 was negative, Ki-67 was less than 2% positive and the mitotic count was between 2-20 per 10 hpf. The staining reactions were confirmatory of a neuroendocrine carcinoma of the pancreas. The final staging of the completely excised tumour was T3 N0 M0. Following discharge, she was followed up regularly and remains symptom free. A CT Scan performed 2 years after surgery (Figure 5) showed no evidence of tumour recurrence.

## Discussion

Pancreatic neuroendocrine tumors (PNETs) are uncommon neoplasms. In Europe and Asia, the reported incidence is < 1 per 100,000 persons per year [1]. No case has been reported from the West African sub-region. Clinically, two groups are identified: functioning and non-functioning neuroendocrine neoplasms. The clinical presentation of functioning tumours depends on the peptide hormone being secreted such as insulin, gastrin, glucagon, vasoactive intestinal peptide (VIP) and somatostatin. Surgical resection for localized disease and selected patients with metastatic disease constitute the primary therapy.

In West Africa, PNETs that are localized to the pancreas and amenable to complete surgical resection are rarely reported. Indeed we found no such report in the English language literature. Ostensibly, many patients present with advanced disease and are not given the benefit of surgical exploration. In the reported case, the patient’s insistence on complete investigation of persistent epigastric pain led to the discovery of the tumour. The pain most likely resulted from stretching of the pancreatic capsule and not from tumour invasion. Because the histological examination indicated complete resection of the tumour, it was judged there was no need for chemotherapy. The patient remains symptom free 2 years after the procedure with no evidence on subsequent imaging of tumour recurrence (Figure 5). The 2010 World Health Organization (WHO) classi@ 257;cation of gastroenteropancreatic neuroendocrine neoplasms (GEP-NENs) and recent genomic data have greatly impacted the clinical management of this disease [2]. The 2010 WHO classification categorizes them into neuroendocrine tumour grade 1 (NET G1), neuroendocrine tumour grade 2 (NET G2) and neuroendocrine carcinoma (NEC) on the basis of the Ki-67 proliferation index and the mitotic count. A mitotic count of < 2 per 10 high-power fields (hpf) and/or a Ki-67 index < 3% corresponds to NET G1; a mitotic count of 2-20/10 hpf and/or a Ki-67 index of 3%-20% to NET G2, and a mitotic count of > 20/10 hpf and/or a Ki-67 index > 20% to NEC [3]. The mitotic count and Ki-67 index are parameters expressing the proliferation rate and the higher of the two is adopted for categorization. Our patient thus fits the descriptors for NET G2. The mitotic count and the Ki-67 index tend to agree in most patients but 44% of pancreatic NET show a discordance hence the need to perform the 2 tests [4]. Although this classification system offers a simple means of categorization, it has been criticized for not considering tumour differentiation and including both well-differentiated and poorly differentiated tumours in the NEC category.

Surgical resection with regional lymph node dissection is the only curative treatment option and is recommended to all patients with early-stage well-differentiated pNENs [3]. Depending on the primary tumour location, the options are: simple enucleation, distal pancreatectomy, with or without splenectomy, central pancreatectomy, pancreatico-duodenectomy (Whipple´s operation) and total pancreatectomy. Patients with NET G1 and G2 generally have a much better prognosis than patients with NEC. Recent studies have reported improved survival after resection or a median overall survival of 58 to 97 months compared to 15 to 21 months in patients not undergoing surgery [5, 6].

## Conclusion

Resectable pancreatic neuroendocrine tumours are rarely encountered in Africa. Thorough investigation of unexplained abdominal pain may lead to early diagnosis which improves the potential for resectability and a good prognosis.

## Competing interests

The authors declare no competing interests.
